# Tool use moves the peri-personal space from the hand to the tip of the tool

**DOI:** 10.3389/fpsyg.2023.1142850

**Published:** 2023-05-12

**Authors:** Ayako Saneyoshi, Ryota Takayama, Chikashi Michimata

**Affiliations:** ^1^Department of Psychology, Teikyo University, Hachioji-shi, Tokyo, Japan; ^2^Department of Psychology, Sophia University, Chiyoda-ku, Tokyo, Japan

**Keywords:** peri-personal space, visual attention, target detection, tool use, extended body

## Abstract

In this study, we used a visual target detection task to investigate three hypotheses about how the peri-personal space is extended after tool-use training: *Addition, Extension*, and *Projection* hypotheses. We compared the target detection performance before and after tool-use training. In both conditions, the participants held a hockey stick-like tool in their hands during the detection task. Furthermore, we added the no-tool-holding condition to the experimental design. In the no-tool-holding condition, a peri-hand space advantage in the visual target detection task was observed. When the participants held the tool with their hands, this peri-hand space advantage was lost. Furthermore, there was no peri-tool space advantage before tool training. After tool training, the peri-tool space advantage was observed. However, after tool training, the advantage of the peri-hand space was not observed. This result suggested that the peri-hand advantage was reduced by simply holding the tool because the participants lost the functionality of their hands. Furthermore, tool-use training improved detection performance only in the peri-tool space. Thus, these results supported the *projection* hypothesis that the peri-personal space advantage would move from the body to the functional part of the tool.

## 1. Introduction

Some studies have reported that the peri-personal space (peri-hand, peri-body, etc.,) is advantageous for many types of cognitive tasks, such as perception (Dufour and Touzalin, 2008), attention (Reed et al., 2006), and memory (Tseng and Bridgeman, 2011). For example, Blini et al. (2018) reported that the visual perception of shapes was more accurate when the objects were presented in the peri-personal space. Similarly, face discrimination was facilitated in peri-personal space (Dureux et al., 2021). Furthermore, Ahsan et al. (2021) submitted that the size of an object was judged more precisely when the object was perceived to be closer to the observer, even when the depth was defined by pictorial cues. Reed et al. (2006) suggested that presenting the participant's hand within the visual field would change the attentional prioritization for the peri-hand space. In Reed et al.'s (2006) study, participants were asked to detect the target while holding their hands thumb-side up with their palms facing toward the center of the visual presentation monitor and their fingertips touching the monitor. The results indicated that the target was detected faster when the target was presented near the hand than when presented far from the hand. Furthermore, this hand presentation effect was observed only when the target was presented near the palm, the functional side of the hands. However, this advantage was not observed when the target was presented near the back of the hand, which is non-functional (Reed et al., 2010; Enomoto and Yamagami, 2011). This result suggests that the hand presentation effect occurred when the target was presented near the *functional* part of the hand.

Previous studies have suggested that the peri-personal space is extended by tool use. Iriki et al. (1996) demonstrated the enlargement of peri-hand space through rake-use training in macaque monkeys. The enlargement was also observed in human studies. Reed et al. (2010) reported that tool use expanded the functional peri-body space. In Reed et al. (2010), participants were asked to detect the target, which was presented in various positions: near the palm, near the prongs of the tool, at the back of the hand, at the back of the tool, and near the forearm. When the target was presented close to the participant's palm or the functional part of the tool after tool training, the detecting performance improved compared to the other positions. In the same vein, some studies suggested that tool use enlarged their perception of reachable space (Witt et al., 2005; Cardinali et al., 2011; Bourgeois et al., 2014). Their results indicated that the functional peri-hand space could be extended through tool-use training.

Several divergent accounts of how the peri-personal space extends to the tool have been proposed. Holmes (2012) suggested three hypotheses: *Extension, Addition*, and *Projection*. The *Extension* hypothesis implies that the functional peri-hand space would be extended from the peri-hand space to the entire part of the tool after tool-use training. The *Addition* hypothesis suggests that the functional part of the tool is added to the peri-hand space after tool-use training. The *Projection* hypothesis insists that the functional peri-hand space moves from the real hand to the functional part of the tool after tool-use training. These three hypotheses differed in the predicted size and position of the attentional fields so that the *Projection* hypothesis could be called the *Substitution* hypothesis. In Holmes et al. (2004), participants held a long-handled tool in each hand. LED light spots were placed near the handle of the tool, the shaft of the tool, and the tip of the tool. In the detection task, tactile stimulation (vibration) was applied to either the thumb or index finger of each hand. They were asked to report the location of the vibrotactile stimulation by pressing the foot pedals. Alongside this tactile stimulation, visual distractors (LED light spots) were presented either above or below on the same or opposite side of the vibrotactile target. The vibration detection performance was interfered with by the incongruent (i.e., on the different side of the vibrated hand) visual stimulus especially near the handle (in peri-personal space). This visual–tactile interaction indicated the peri-personal space advantage for detecting the target. During the detection task, they sometimes performed tool training in which they used the tool to press a button located above the tabletop and away from the participant. They pressed the randomly illuminated LED on the buttons with the designated screws on the tool. Furthermore, this visual–tactile interaction was modulated by the parts of the tools used to perform the tool training task. For example, the condition in which they used the tip of the tool showed visual–tactile interaction at the tip of the tool as well as near their hands, whereas this improvement was not observed in the shaft of the tools. This result suggests that the peri-hand space was added only around the functional part of the tool. Hence, they proposed that this result supported the *Additional* hypothesis. By contrast, Bonifazi et al. (2007) proposed the *Extension* hypothesis. They investigated the three hypotheses based on the “visual tactile extinction” of participants with brain damage. In their studies, the visual and tactile stimuli were presented to the left and right sides of the participant's body. Owing to the brain damage, the perception of the tactile stimulation on the contralesional side of the body is erased when the visual stimulus is presented on the ipsilateral side (visual–tactile extinction). Although this phenomenon was observed when the visual stimulus was presented near the body parts, the space which produced extinction was extended to the tip of the tool and the shaft of the tool after the tool training task. Hence, they suggested that the peri-hand space was entirely extended by the tool use from the hand to the tip of the tool. Therefore, these data supported the *Extension* hypothesis.

However, Park and Reed (2020) insisted that these conflicting results stemmed from the differences in the way the tool was used and the space it was used. Park and Reed (2020) suggested that these different tool actions would produce different types of peri-personal space around the tool. In the Holmes et al. (2004) task, the buttons in the training task were all located away from the participant's body, and participants simply pressed the button with the functional part of the tool away from their body, so there was no need to remap the peri-hand space to the entire tool. Contrarily, in Bonifazi, Farnè, Rinaldesi and Làdavas (2007) task, participants had to rake the object from a far to a near space using the entirety of the rakelike tool. In this task, they had to pay attention to the entire tool and the tool-holding hand; thus, the peri-personal space was remapped to the entire tool and hand. In Park and Reed (2020) study, participants used the different parts of the tool during tasks, and their results showed that peri-personal space would be remapped to the tooltip or extended to the whole tool depending on the task and the part of the tool the participants had to use to perform the task.

With regard to previous studies (Reed et al., 2010; Enomoto and Yamagami, 2011), the peri-body advantage space should be limited to around the functional part of the body. Furthermore, when gripping the tool shaft, the hand lost its function and peri-hand space advantage. However, few studies suggested the *Projection* hypothesis. Moreover, there were two problematic factors in the methods used in the previous studies. First, previous studies used visual tactile stimulation in their tasks (Holmes et al., 2004; Bonifazi et al., 2007; Park and Reed, 2020). Tactile stimuli to the hand may keep attention focused on one's hands, which retained the advantage for peri-hand space. In fact, Geers and Coello (2023) suggested that tactile stimulation would facilitate the integration with the visual information in peri-personal space to prepare for the action. Second, the pre-tool practice conditions (i.e., control condition) were generally measured while gripping the tool (Park and Reed, 2020). As the tool was gripped, the peri-hand space advantage could have been compromised already. Hence, the performance of peri-hand space did not change before and after tool-use training in the previous studies.

In this study, we used a simple visual target detection task but not a visual–tactile interaction task to investigate how peri-personal space extended to the tool. Furthermore, we compared the detection performance in the peri-hand space between the conditions without the tool, holding the tool before and after training. If the peri-personal space enlarged around the functional part of the tool and body, the target detection performance near the hand would be faster than far from the hand only when they were not occupied with the tool. Furthermore, this advantageous space would enlarge to include the functional part of the tool after tool training.

## 2. Methods

### 2.1. Participants

In total, 26 undergraduate students (12 females, mean age = 20.16 years, SD = 2.07 years) volunteered to participate. We calculated the sample size using the G^*^Power software (Faul et al., 2007). For a moderate effect size, power of 0.80, and *p-value* of 0.05, we needed a sample size of at least 24. All participants had normal or corrected-to-normal visual acuity. They were unaware of the hypothesis under investigation. Before participation, they provided informed written consent. All experimental procedures were approved by the Institutional Review Board of the Department of Psychology of Teikyo University.

### 2.2. Experimental design

The experimental design was an orthogonal combination of three tool conditions (no tool–pre-tool training, with tool–pre-tool training, and with tool–post-tool training) and three target positions (near hand, near tool, and far from both). In addition, the target was projected on the left or right side of the tool and hand. All the variables were manipulated among participants. The dependent variable was reaction time (RT) to detect the target. If the *Projection* hypothesis was appropriate, the RT of the near-hand condition would be shorter than that of the near-tool condition and far conditions in the no tool–pre-training condition, whereas, in the with tool–pre-training condition, the advantage of the near-hand condition would be lost. Moreover, in with tool–post training condition, the RT of the near-tool condition would be shorter than that of the near-hand condition. If the *Addition* or *Extension* hypothesis was appropriate, we would expect to see a near-hand condition advantage in both no tool–pre-training and with tool–pre-training conditions. Furthermore, no difference in RT between the near-tool and near-hand conditions would be predicted in the with tool–post-training condition.

### 2.3. Apparatus

All stimuli were projected on the white tabletop (60 cm x 90 cm space) by the LCD projector (QUMI Q5, Vivitek) connected to a laptop computer (MacBook Pro, Apple) running Matlab R2016a (MathWorks) with Psychtoolbox (Brainard, 1997). A keyboard was connected to the computer and served as a response console.

**Figure 1:**
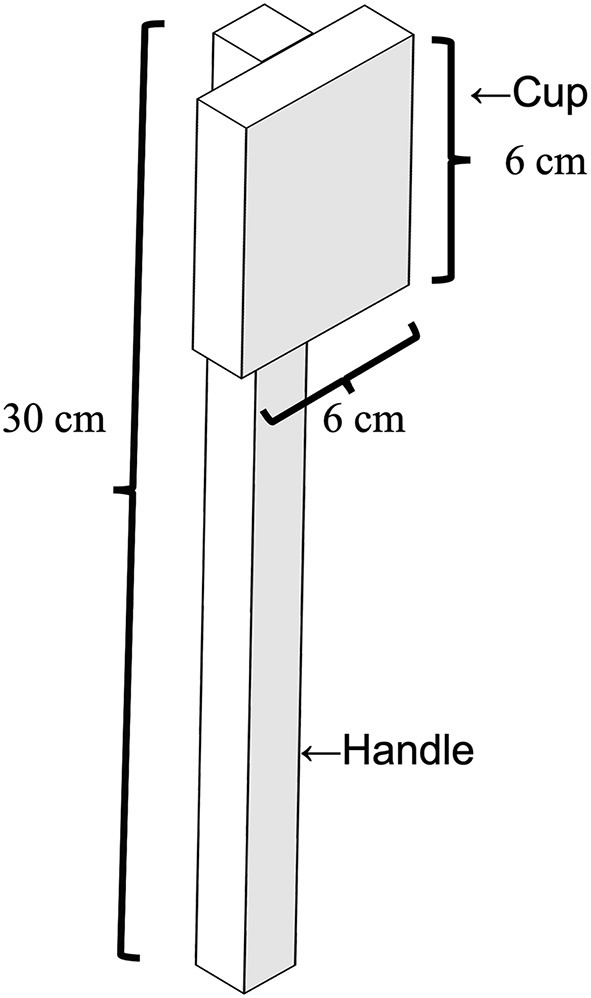
The wooden stick tool used in the hockey-like tool training game.

**Figure 2:**
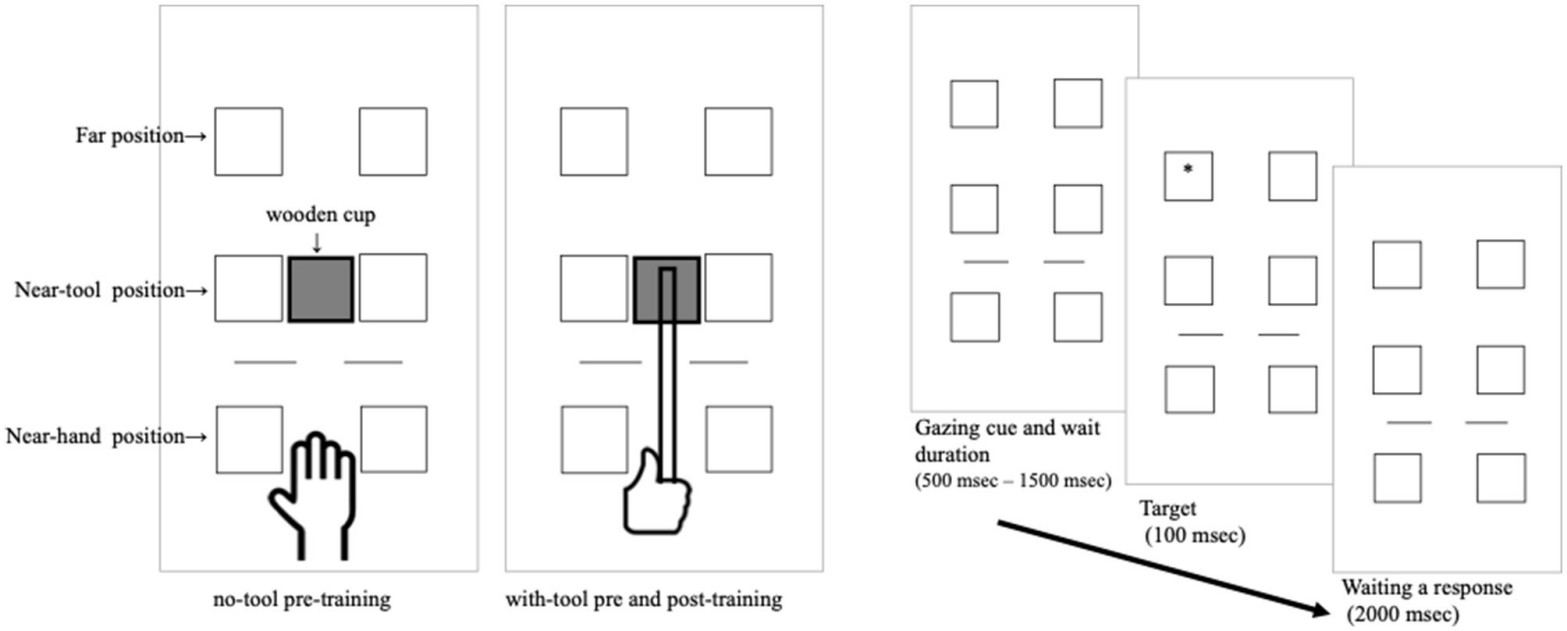
Procedure for the detection task. The left side indicates the no-tool condition and the right side the with-tool condition.

We prepared the wooden items for the hockey-like game task, which comprised a stick, puck, and obstacle disks. The wooden stick consisted of a balsa handle (30 cm in length) with a square wooden cup (6 × 6 cm) attached to the end (see Figure 1). The puck was a wooden disk (4 cm in diameter and 1 cm in height). There were 11 small wooden obstacle disks and an L-shaped wooden goal at one end of the course. The course space was a 29.7 × 84-cm whiteboard on the table (see Figure 2).

### 2.4. Stimuli

There were six black line boxes (3 × 3 cm) on the table during the experiment. The boxes were presented symmetrically on the left and right sides of the center line. The distance from the center of the boxes was 5 cm from the center line. The target was presented in each of these boxes. There were three positions where the target was presented. In the far condition, the target was presented 90 cm from the participants. In the near-tool cup condition, the target was presented at the position where the tool cup was set, 60 cm from the participants. In the near-hand condition, the target was presented at the position where the hand was set, 30 cm from the participants. The boxes were always presented. The target was a black asterisk. The stimulus and the gazing cue were presented in black on the white table (see Figure 2).

### 2.5. Task and procedure

First, the participants performed the detection task (no tool–pre-training), followed by the second detection task (with tool–pre-training). Subsequently, they performed the tool-use practice (hockey-like game) before the third detection task (with tool–post-training).

#### 2.5.1. Detection task

The participants were seated in front of the table. They were asked to sit close to the table and put their hands in the specified position, ~30 cm from the edge of the table. They were told to maintain their seated position and posture during the task. In the no-tool condition, the wooden board (6 × 6 cm), which was the same as the cup of the hockey-like wooden tool, was set in the position where the tool cup was placed in the tool-holding condition. There was the possibility that the presence of an object might attract visual attention in the detection task. By this manipulation, we attempted to reduce the effect of the board's presence in the condition. In the pre-tool training and post-tool training conditions, they had to hold the wooden tool that was used in training (hockey-like game) with their right hand during the task. There were 84 trials (including 12 catch trials) in each condition. In a trial, first, two horizontally aligned 3-cm lines were presented as the gazing cue on the table at a distance of 45 cm from the participant's body. After the gazing cue was presented for a randomly selected duration time between 500 and 1,500 msec, the target was presented. In order to keep the participant's attention on the task, the duration between the presentation of the gazing cue and the presentation of the target was randomized. The target was presented in one of the six boxes for 100 msec. The target was presented 12 times for each of the six boxes (36 times on each side, left and right), and participants were instructed to keep their gaze on the gazing cue.

#### 2.5.2. Tool training: “Hockey-like game”

**Figure 3:**
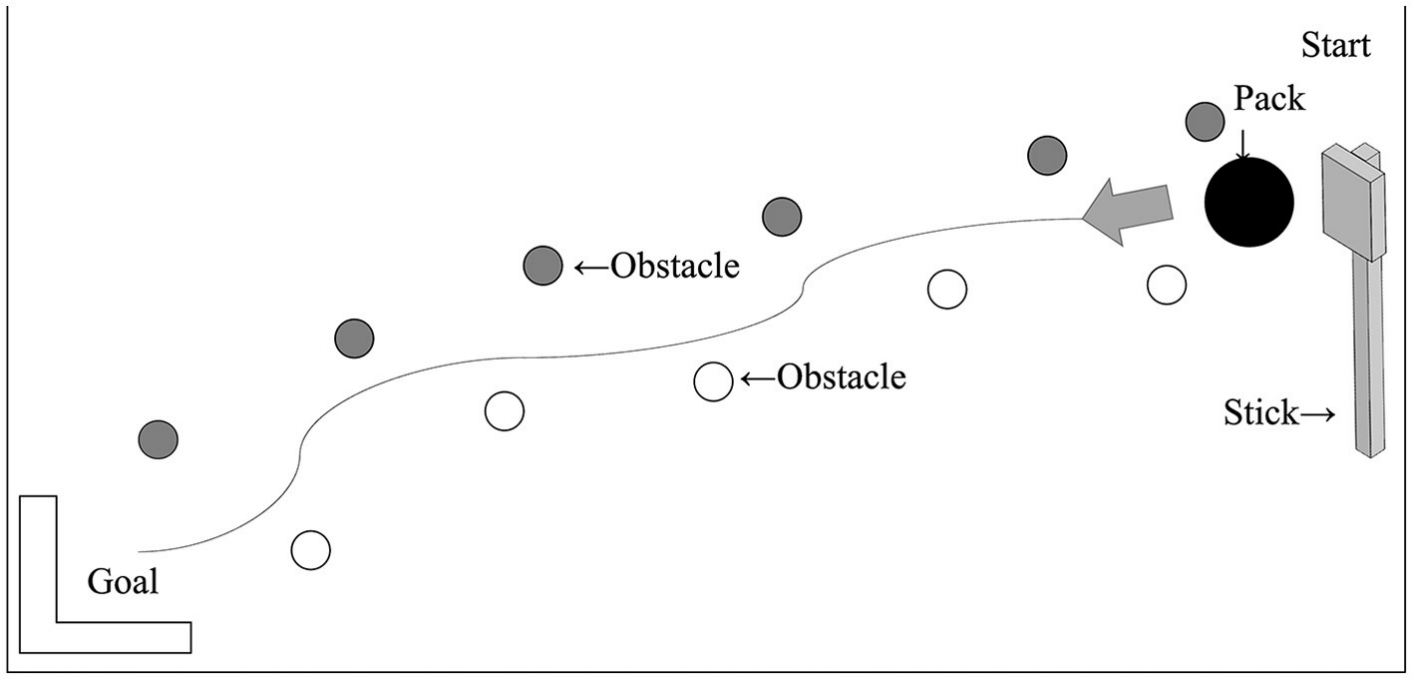
The course of the hockey-like game. The gray and white disks represent the obstacle disks. The participants tap the side of the puck and slide it between the gray and white obstacle disks. The large disk represents the puck, and the L shape represents the goal.

The tool-use training task was a hockey-like game (Park and Reed, 2015). There were 11 objects on the course space (29.7 × 84 cm, whiteboard). The participants used the wooden tool through the obstacles (see Figure 3). The participant stood in front of the board with the wooden tool stick. They were instructed to tap the side of the puck with the cup part of the wooden tool stick from the upper right starting position and slide it to the L-shaped goal in the lower left of the board. They were to move the puck without hitting any obstacles. The participants were asked to repeat the task as many times as possible within 3 min and could only touch the puck with the cup on the tool.

## 3. Results

For the hockey-like game, the average number of goals was 14.48 (SD 3.63). This number of goals was comparable to a previous study (Park and Reed, 2015) in which they reported a significant training effect. Thus, participants received sufficient training and experience for tool use.

The average error rate for the catch trial was 0.36%, indicating that participants performed the task correctly. To determine whether there were left–right differences in target presentation position, a repeated-measures ANOVA was performed for RT with three factors: left–right position x target position x tool condition. There was no interaction related to the left–right position, *F* (4,100) =0.56, *p* =0.696, ${\text{η}}_{\text{p}}^{2}$ = 0. 02. Therefore, we combined both data for the left and right target positions in the following analysis.

**Figure 4:**
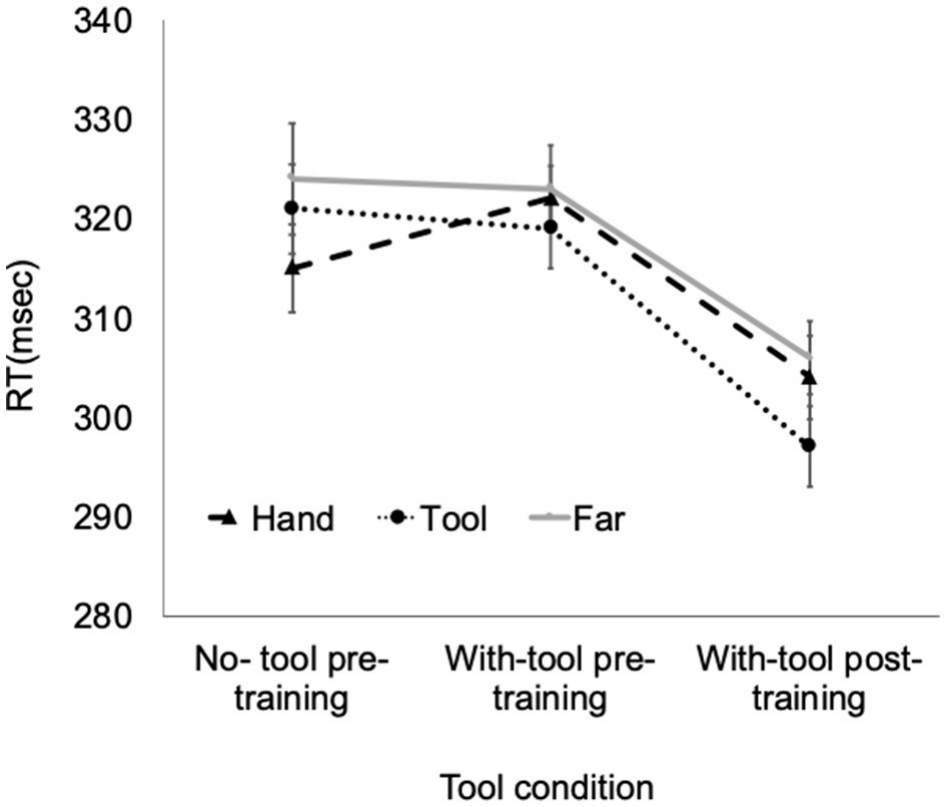
Result of Experiment 2. The error bars represent the standard error of means.

A repeated-measures ANOVA was performed for RT with-tool condition (3: no tool–pre-training, with tool–pre-training, and with tool–post training) x target position (3: near hand, near tool, and far). Before the ANOVA, we confirmed the sphericity assumption of data by Mauchly's test and the normality of the data by Kolmogorov–Smirnov test. There was a main effect of tool condition, *F*_(2,50)_ = 14.69, *p* < 0.001, ${\text{η}}_{\text{p}}^{2}$ = 0.37 and target position, *F*_(2,50)_ = 3.57, *p* = 0.036, ${\text{η}}_{\text{p}}^{2}=$ 0.12. In addition, there was an interaction in tool condition x target position, *F*_(4,100)_ = 4.58, *p* = 0.002, ${\text{η}}_{\text{p}}^{2}$ = 0.15. A one-way ANOVA for RT by target position was performed in each tool condition. There was a significant main effect of target position in the no tool–pre training condition and with tool–post-training condition. In the no tool–pre-training condition, the RT of the near-hand condition (mean = 315 msec, SD = 39 msec) was significantly shorter than the near-tool (mean = 321 msec, SD = 43 msec) and far conditions (mean = 324 msec, SD = 47 msec), near hand–near tool: *p* = 0.043, near hand–far: *p* = 0.009. This near-hand advantage was reversed in the with tool–post-training condition, near hand (mean = 304 msec, SD = 37 msec)–near tool (mean = 297 msec, SD = 37 msec): *p* = 0.007; near tool–far (mean = 306 msec, SD = 38 msec): *p* = 0.005. There was no significant main effect of target position in the with tool–pre-training condition, *F*_(2,50)_ = 14.69, *p* < 0.001, ${\text{η}}_{\text{p}}^{2}$ = 0.37 (near hand: mean = 322 msec, SD = 47 msec; near tool: mean = 319 msec, SD = 42 msec; far: mean = 323 msec, SD = 44 msec) (see Figure 4).

## 4. Discussion

In this study, we investigated how tool training would change the peri-hand space for a visual target detection task. In the no tool–pre training condition, a peri-hand space advantage in the visual target detection task was observed. When the participants held the tool with their hands, this peri-hand space advantage was lost. In other words, simply gripping the tool did not produce an advantage for the peri-tool space. This finding was consistent with that of Witt et al. (2005), who reported that perceived reachable space enlarged only when the observer intended to use the tool. Furthermore, there was no peri-tool space advantage in with tool–pre-training condition. After tool training, the peri-tool space advantage was observed. However, after tool training, the advantage of peri-hand space was not observed. This result suggested that the peri-hand advantage was reduced simply by holding the tool, as the participants lost the functionality of the hand (holding, touching, etc.).

Furthermore, tool-use training improved the detection performance only in the peri-tool space. In previous studies that reported peri-hand space advantage, the improvement in target detection performance was observed only when the target was presented close to the functional part of the hand (Reed et al., 2006, 2010; Enomoto and Yamagami, 2011). These results suggested that the loss of function led to the decline of the attentional advantage of the peri-hand space. Furthermore, after the tool-use training, this superior space for detection was moved to the functional part of the tool. These results supported the *Projection* hypothesis, in which the tool-use training moves the peri-hand space from the real hand to the functional part of the tool.

Some previous studies support the *Addition* and *Extended* hypotheses (Holmes et al., 2004; Bonifazi et al., 2007). In other words, the peri-hand space always maintained its advantage. However, the results of this study showed that the peri-hand advantage was also lost when the hand lost function by gripping the tool, in comparison with the no-tool condition in which the hand did not hold the tool. In addition, the peri-hand space moved to the functional area of the tool when participants became proficient with the tool through the training received. What explains the difference between the results of previous studies and this study? Park and Reed (2020) insisted that the conflicting results over the three hypotheses stemmed from the differences in the way the tool was used and the space it was used in. In the hockey-like training in this study, participants were asked to manipulate the tool delicately and pay attention to the functional part of the tool. Therefore, the peri-personal space was remapped to the tool. However, as hand function was lost by gripping the tool, peri-personal superiority space was lost. In accordance with Park and Reed (2020), the peri-personal space was limited to the functional part of the tool after the training task. Hence, our results seemed to be congruent with the *Projection* hypothesis.

In addition to the claims of Park and Reed (2020), there was a difference in the task procedures between the previous studies and this study. de Vignemont and Iannetti (2015) proposed two types of peri-personal space: one was for defensive action, and the other was for working action. Although there is some debate as to whether the two types of peri-personal spaces are independent or the same representation is used (de Vignemont and Iannetti, 2015), multiple types of peri-personal space should exist. In this study, the stimulus and the detection task were simple and neutral, and thus, they would not evoke the defensive type of peri-personal space. By contrast, in the previous studies, participants were asked to discriminate the location of a tactile stimulation (Holmes et al., 2004; Bonifazi et al., 2007; Park and Reed, 2020). It has been suggested that passive tactile stimulation to the hand or body would indicate a dangerous impression (Fossataro et al., 2016). Therefore, it may be possible that the task with tactile stimulation triggered the peri-personal space with a defensive mode. There was a possibility that in defensive mode, the hand would not lose peri-personal space advantage even after losing function by grasping the tool. However, it is not yet known whether the different types of peri-personal space correspond to the three types included in the peri-hand space hypothesis, and further studies are necessary.

Recently, a study reported that the peri-personal space had been extended to a remote-controlled hand avatar in a virtual environment (Mine and Yokosawa, 2021). Using an immersive head-mounted display, the participants observed the hand avatar presented at a position far from their real hand. After grasping experience with the avatar hand, the participants showed visuo-tactile facilitation of the far remote hand, while there was no facilitation in the near space around the real hand. This result would support the *Projection* hypothesis.

In this experiment, we placed the wooden board in the position where the tool cup was placed in the tool hold condition. In the result, even when the target was presented near this wooden board, the detection speed was not faster than that of the far condition. This result suggested that the facilitation of the target detection would not be according to the visual anchors but according to the functional hand or tool presence.

In this study, the detection task was performed three times: no tool–pre training, with tool–pre-training, and with tool–post-training. Therefore, there is a practice effect in our results. In fact, the overall detection RT was becoming faster with each task. Consequently, the improvement of detection performance in the peri-tool space after the tool training could be due to the repetitive practice effect. However, a simple practice effect could not explain the loss of advantage in the peri-hand space in with tool–pre-training condition and the emerging advantage only for the peri-tool space after tool training. These tool conditions and target position interaction indicate that the peri-hand advantage space moved with-tool training.

We found that the peri-personal space is set up on the functional space of the tool and body. Therefore, if the hand loses function, the advantage of peri-personal space disappears. Once individuals become proficient in using the tool, the advantage area moves to the functional parts of the tool. Furthermore, visual attention was broader in this enlarged space, and the detection performance improved. This study provides insights into how people become proficient tool users and demonstrates the flexibility of the peri-personal space.

## Data availability statement

The raw data supporting the conclusions of this article will be made available by the authors, without undue reservation.

## Ethics statement

The studies involving human participants were reviewed and approved by Ethical Committee for Human Psychological Research of Teikyo University. The patients/participants provided their written informed consent to participate in this study.

## Author contributions

AS and RT developed and conducted the experiments, developed the statistical analysis plan, and conducted statistical analyses. AS drafted the original manuscript. CM supervised the conduct of this study. All authors approved the final version of the manuscript to be published, conceived the idea of the study contributed to the interpretation of the results, and reviewed the manuscript draft and revised it critically on intellectual content.
